# First molecular detection and genetic characterization of *Coxiella burnetii* in Zambian dogs and rodents

**DOI:** 10.1186/s13071-018-2629-7

**Published:** 2018-01-17

**Authors:** Simbarashe Chitanga, Edgar Simulundu, Martin C. Simuunza, Katendi Changula, Yongjin Qiu, Masahiro Kajihara, Ryo Nakao, Michelo Syakalima, Ayato Takada, Aaron S. Mweene, Samson Mukaratirwa, Bernard M. Hang’ombe

**Affiliations:** 1 Department of Biomedical Sciences, School of Health Sciences, University of Zambia, PO Box 50110, 10101 Lusaka, Zambia; 2 Department of Disease Control, School of Veterinary Medicine, University of Zambia, PO Box 32379, 10101 Lusaka, Zambia; 3 Department of Paraclinical studies, School of Veterinary Medicine, University of Zambia, PO Box 32379, 10101 Lusaka, Zambia; 4 Hokudai Center for Zoonosis Control in Zambia, School of Veterinary Medicine, University of Zambia, PO Box 32379, 10101 Lusaka, Zambia; 5 Division of Global Epidemiology, Hokkaido University Research Center for Zoonosis Control, Sapporo, Japan; 6 Unit of Risk Analysis and Management, Hokkaido University Research Center for Zoonosis Control, Sapporo, Japan; 7 Laboratory of Parasitology, Department of Disease Control, Graduate School of Veterinary Medicine, Hokkaido University, Sapporo, Japan; 8 Department of Animal Health, School of Agricultural Sciences, Northwest University, Mafikeng Campus, P/B. X2046, Mmabatho, South Africa; 9 School of Life Sciences, University of KwaZulu-Natal, Durban, South Africa

**Keywords:** *Coxiella burnetii*, Domestic dogs, Rodents, Phylogenetic analysis, Zambia

## Abstract

*Coxiella burnetii*, the causative agent of Q fever, is a zoonotic pathogen associated with sylvatic or domestic transmission cycles, with rodents being suspected to link the two transmission cycles. Infection and subsequent disease in humans has historically been associated with contact with infected livestock, especially sheep. However, recently there have been reports of Q fever outbreaks associated with contact with infected rodents and dogs. Studies exploring the potential role of these animal hosts in the epidemiology of Q fever in many developing countries in Africa are very limited. This study aimed to determine the potential role of rodents and dogs in the epidemiological cycle of *C. burnetti* in Zambia. Using pathogen-specific polymerase chain reaction assays targeting the *16S* rRNA gene, *C. burnetii* was detected for the first time in 45% of rodents (9/20), in one shrew and in 10% of domestic dogs (15/150) screened in Zambia. Phylogenetic characterization of six samples based on the isocitrate synthase gene revealed that the strains were similar to a group of isolates from chronic human Q fever patients, goats and rodents reported in multiple continents. Considering the close proximity of domestic dogs and rodents to humans, especially in resource-limited communities, the presence of *C. burnetii* in these animals could be of significant public health importance. It is thus important to determine the burden of Q fever in humans in such resource-limited communities where there is close contact between humans, rodents and dogs.

## Letter to the Editor

*Coxiella burnetii*, the causative agent of the zoonotic disease Q fever, is assumed to have a sylvatic and a domestic transmission cycle in which livestock play the most important role, with rodents suspected to act as a link between the two cycles [1]. Human infection is characterized by febrile illness and community-acquired pneumonia that may become chronic, resulting in mortalities in few cases [2]. In livestock, the disease is mainly asymptomatic, but has been associated with abortions and decreased livestock productivity, with consequent negative socioeconomic effects on livestock farmers [2].

Whilst there have been no reported cases of Q fever in humans in Zambia, serologic evidence in humans has been reported in areas where they co-exist with livestock [3]. Moreover, the presence of *C. burnetii* in Zambian livestock has been genetically confirmed [4]. However, the potential role of rodents and domestic dogs in the epidemiology of Q fever in Zambia has not been explored, despite the observed association between these hosts and disease outbreaks in humans in other countries [1, 5]. This study aimed to detect and characterize *C. burnetii* in rodents and semi-confined domestic dogs from rural and peri-urban settings, respectively, to ascertain the potential role of these animal hosts in the epidemiological cycle of this pathogen.

In 2012–2013, blood and internal organs (spleen, lungs, heart and liver) were sampled from 20 rodents (*Mastomys natalensis*: *n* = 11; *Gerbillinae* sp.: *n* = 7; *Saccostomus campestris*: *n* = 2) and one shrew trapped from Nyimba (*n* = 9) and Namwala (*n* = 12) Districts in Zambia. The blood and internal organs from each rodent were pooled and homogenized. One-hundred-and-fifty blood samples were collected from dogs from 2016 to 2017 in Chilanga District [Mwembeshi (*n* = 86) and Mapepe (*n* = 64)]. Nyimba and Namwala Districts are plague-endemic rural areas [6] whereas Chilanga District is mostly peri-urban.

DNA was extracted from canine blood and homogenates of blood and internal organs of rodents. For molecular detection, the primers QR-FO (5′-ATT GAA GAG TTT GAT TCT GG-3′) and QR-RO (5’-CGG CCT CCC GAA GGT TAG-3′), which amplify a 1450 bp fragment of the *16S* rRNA gene of *C. burnetii*, were used [7]. *Coxiella*-positive samples were further characterized by amplification of a 738 bp fragment of the *C. burnetii-*specific isocitrate dehydrogenase (*icd*) gene using the primer pair *icdtrg_f* (5′-CGG AGT TAA CCG GAG TAT CCA-3′) and *icdtrg_r* (5′-CCG TGA ATT TCA TGA TGT TAC CTT T-3′) [8]. Purified polymerase chain reaction products of the *icd* gene were sequenced directly by the Sanger method using a 3130 genetic analyzer. The sequences obtained (four from rodents and two from dogs) were deposited in the GenBank database (accession numbers LC319605–LC319610) and utilized in the phylogenetic analyses.

Overall, the shrew, 45% of rodents (9/20) and 10% of dogs (15/150) screened were positive for *C. burnetii*. Nyimba District had a prevalence of 11.1% (1/9) whilst for Namwala, the prevalence was 75% (9/12) in rodents. The detection among the rodents was as follows: *Saccostomus campestris* (2/2), *Gerbillinae* sp. (4/7) and *Mastomys natalensis* (3/11). The prevalence in dogs by sampling sites was 10.9% for Mwembeshi and 9.3% for Mapepe.

**Fig. 1:**
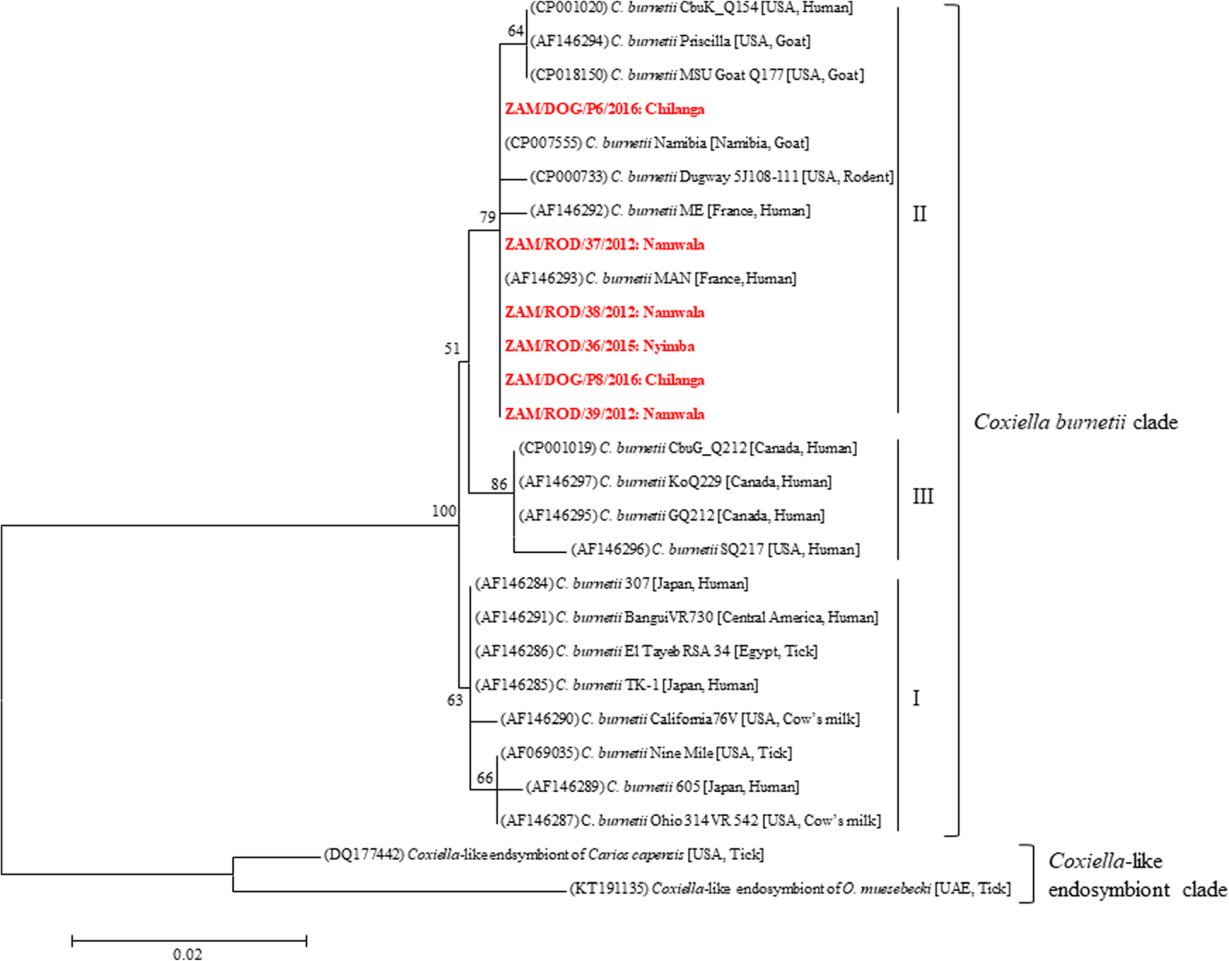
Evolutionary relationships of *Coxiella* strains based on isocitrate dehydrogenase (*icd*) gene sequences. The neighbor-joining tree was constructed using Kimura 2-parameter model in MEGA software. The GenBank accession numbers are in parentheses while the host and country of origin are in square brackets. The *Coxiella* strains characterized in this study are in bold and red text, with the district of origin indicated after the colon. Bootstrap values ≥ 50% are shown at branch nodes. The scale-bar indicates the number of substitutions per site

Nucleotide BLAST comparison (https://blast.ncbi.nlm.nih.gov/Blast.cgi) revealed that all the Zambian *C. burnetii icd* gene sequences showed highest similarity (99%) to that of *C. burnetii* strain MAN (GenBank: AF146293), which was isolated from a chronic human case of Q fever in France [9]. Phylogenetic analysis of the *icd* gene sequences revealed that *C. burnetii* isolates separated into three main groups (Fig. 1). Group I consisted of isolates from acute human Q fever patients, ticks and cows. Group II included isolates from chronic human Q fever patients, goats, rodents and dogs (Fig. 1). The Zambian strains of *C. burnetii* belonged to “chronic” group II, intimating possible long-term circulation of the pathogen in these hosts. Group III consisted of chronic human Q fever isolates found in Canada and the USA.

To the best of our knowledge, this is the first report of molecular detection and characterization of *C. burnetii* in rodents and semi-confined domestic dogs in Zambia. Moreover, studies in these animal hosts are very limited in many developing countries in sub-Saharan Africa. In most rural and peri-urban communities of Zambia, humans, especially children, are in contact with the environment co-shared with rodents and dogs, a factor which could predispose them to infection through inhalation of contaminated environmental dust [10]. Since the dogs sampled were semi-confined, the risk of infection potentially extends to a wider community. Furthermore, as the rodents sampled were trapped in plague-endemic areas [6], there is a possibility that individuals living in these communities could be co-infected by both pathogens. Along with the fact that *Plasmodium falciparum* malaria is endemic in Zambia and presents with similar clinical signs, these scenarios could pose serious challenges with regards to diagnosis and patient management in resource-limited settings.

Our findings demonstrated the circulation of *C. burnetii* in rodents and domestic dogs, highlighting the potential threat this pathogen poses to humans. This emphasizes the need for more extensive studies to determine the burden of Q fever in humans in communities where rodents and dogs have tested positive and in other regions of the country to determine the reservoir role of these animals.

## Abbreviations

*Icd*: Isocitrate dehydrogenase
rRNA: Ribosomal ribonucleic acid
